# Effects of Steelmaking Slag and Moisture on Electrical Properties of Concrete

**DOI:** 10.3390/ma13122675

**Published:** 2020-06-12

**Authors:** Se-Hee Hong, Tian-Feng Yuan, Jin-Seok Choi, Young-Soo Yoon

**Affiliations:** School of Civil, Environmental and Architectural Engineering, Korea University, 145 Anam-ro, Seongbuk-gu, Seoul 02841, Korea; bestshhong@korea.ac.kr (S.-H.H.); yuantianfeng@korea.ac.kr (T.-F.Y.); radiance@korea.ac.kr (J.-S.C.)

**Keywords:** steelmaking slag, setting times, compressive strength, electrical resistivity, electrical piezoresistivity, electrical sensitivity

## Abstract

Strain sensors can indicate the conditions of concrete structures, but these sensors are only capable of measuring local behaviors of materials. To solve this problem, researchers have introduced conductive materials that can monitor the overall behavior of concrete structures. Steelmaking slag, which contains large amounts of iron oxide (Fe_2_O_3_), is conductive, and researchers have considered the addition of this material to improve concrete monitoring. In this study, mechanical and electrical properties of concrete containing steelmaking slag as a binder were evaluated. As the incorporation of steelmaking slag increased, the setting times were delayed, but the compressive strengths were similar within the replacement ratio of 15%. It was found that the addition of steelmaking slag with Fe_2_O_3_, the main ingredient of magnetite (Fe_3_O_4_), improved the electrical resistivity, piezoresistivity, and sensitivity of the concrete. Drying of the concretes resulted in an increase in electrical resistance and fractional change in resistivity (FCR). Expansion of steelmaking slag, due to contacting of free CaO and moisture under repeated loads, resulted in cracks in the concrete and affected the gauge factor (GF). This study demonstrates the possibility that the addition of steelmaking slag as a binder may provide an economical and environmentally-friendly solution to concrete strain monitoring.

## 1. Introduction

As concrete technology improves, concrete structures are lasting longer and are subject to material deterioration and changing environments, including climate [1,2,3]. Environmental and structural stressors shorten the life of concrete. Therefore, there is an increasing interest in the development of efficient, economic, and accurate methods to assess the structural health of concrete structures [1,4]. Conventional and commercial strain sensors for infrastructure can only assess the local behavior of structures, and they are limited in use due to poor durability, low sensitivity, and low gauge factors [1,4,5,6,7]. To address the need for accurate and practical concrete monitoring methods, researchers have proposed “smart concrete structures” that measure load/deformation and stress/strain by incorporating conductive materials [2,3,4].

Concrete and cement composites become sensible by incorporating special functional fibers and fillers. It is well-known that incorporation or replacement ratio of conductive materials, distribution states of fillers, and types of additives strongly influence the conductivity of cement composites and concretes. Choi et al. [4] found that the incorporation of 0.75% steel fibers into concrete resulted in an effective conductive path while entangling each other in the concrete matrix. Lee et al. [8] assessed the effect of carbon fibers on the electrical properties of cement composites and found that incorporating ratios of carbon fibers that exceeded 1% increased the porosity of the cement composites. They concluded that it is desirable to incorporate 0.5–1.0% of carbon fiber. Functional fillers used in concrete or cement composites typically include carbon nanotubes (CNTs) and carbon black. Lee et al. [8] and Yoo et al. [9] evaluated the piezoresistivity by incorporating 1% of multi-walled carbon nanotubes (MWCNTs) into cement composites. They found that, when an external load was applied to these cement composites, MWCNTs were close to each other, resulting in an excellent conductive path due to tunneling effects. The gauge factors of cement composites containing MWCNTs in these studies were 166.6 and 113.2, respectively [8,9]. In a different study, Monteiro et al. [10] suggested that the optimal amount of carbon black to improve both mechanical and electrical properties of concrete containing is 7%. Together, these studies demonstrate that the incorporation of conductive materials into concrete may enable structural health monitoring (SHM) and vehicle detection of civil infrastructure [1,3,11].

Meanwhile, recently, high-strength reinforcing bars have been developed and manufactured to improve structural performance of concrete structures. As the production of high-strength reinforcing bars increases, so does the production of steelmaking slag, a steel byproduct for which it is difficult to secure a site for aging and landfilling [12]. However, a recent study found that the incorporation of steelmaking slag as a fine aggregate made cement composites electrically conductive, which can be used as strain sensors [13]. Electrically conductive characteristic of cement composite containing steelmaking slag as a fine aggregate enables the durability evaluation and safety diagnosis of high-rise buildings and nuclear power plants through SHM [14].

The substitution of cement to steelmaking slag may also decrease the amount of carbon dioxide (CO_2_) generated in concrete and construction fields. The production of one ton of ordinary Portland cement (OPC) generates 1 ton of CO_2_, and it is estimated that 7% of the worldwide CO_2_ generation can be attributed to the cement industry [15]. Lee et al. [16] reported that, even when steelmaking slag replaced 15% of the cement weight in a concrete, there was no significant decrease in strength compared to ordinary concrete. In addition, Hong et al. [12] evaluated the microstructure and strength characteristics of concrete that included steelmaking slag and found that the concrete performance deteriorated when the proportion of steelmaking slag exceeded 20%. These studies indicate that the incorporation of steelmaking slag as a supplementary cementitious material may increase the conductivity of the material, reduce the environmental impact of the production of concrete, and provide a durable and strong material that could be used in SHM.

In this study, it was investigated that how the replacement ratio of steelmaking slag changed the mechanical (setting time and compressive and splitting tensile strength) and electrical (resistivity, piezoresistivity, and sensitivity) properties of concrete. Because moisture in concrete is reported to have a significant effect on the electrical properties of concrete, the electrical properties of the concrete with steelmaking slag was also compared before and after drying.

## 2. Experimental Program

### 2.1. Mix Designs, Materials, and Specimen Preparation

The mix design of the concrete specimens used in this study are shown in Table 1. Hong et al. [12] evaluated the microstructure and strength characteristics of concrete containing steelmaking slag according to water to binder ratio (w/b). Among the three w/b (0.275, 0.300, and 0.325), the best performance was shown in 0.325. Therefore, in this study, w/b was fixed at 0.325 and the replacement ratio of steelmaking slag was set to 0%, 10%, 15%, and 20% of the cement weight. On the other hand, some studies have reported that, due to the low hydraulic characteristic of steelmaking slag, the strengths of the concrete at the early age decreases as the replacement ratio of steelmaking slag increases [16,17]. This characteristic may affect the strength development of concrete containing steelmaking slag. Therefore, experiments were performed on five mixes in consideration of gypsum (calcium sulfate hemihydrate gypsum) incorporation in order to prevent drop of strength caused by steelmaking slag incorporation which was replaced as a binder.

**Figure 1:**
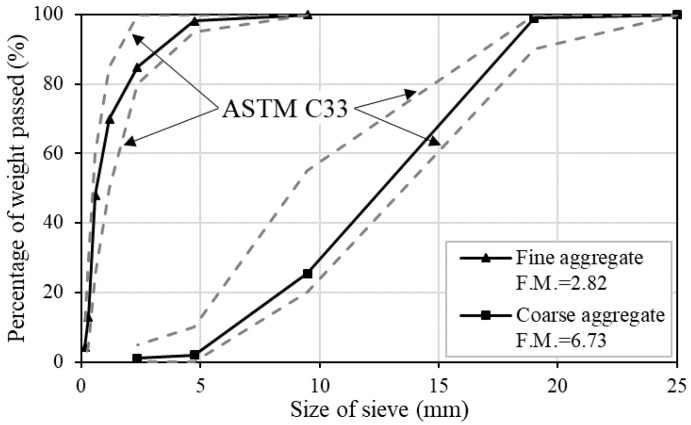
Sieve analysis of the aggregates.

Table 2 summarizes the chemical and physical characteristics of the cementitious materials used in this study. As cementitious materials, Type I OPC and steelmaking slag were used. Steelmaking slag has varying properties according to the steelmaking process, and, in this study, electric arc furnace oxidizing slag containing approximately 40% of iron oxide was used (Fe_2_O_3_). Steelmaking slag was first crushed with a jaw crusher, and then a ball mill was used to make steelmaking slag aggregate into powder. It was grounded by a ball mill for 3 h and the blaine of steelmaking slag reached approximately 5000 cm^2^/g. Particle size distribution was analyzed through CILAS 990, and steelmaking slag had a mean diameter of 27.15 μm. Both cementitious materials and aggregates were produced in Republic of Korea. River sand was used as fine aggregate, and the coarse aggregate had a maximum dimension of 19 mm. To determinate the size of aggregates, sieve analysis was performed according to ASTM C136 [18]. Figure 1 represents the particle size distribution curves of the aggregates and it satisfied the ASTM C33 [19]. The fineness modulus (F.M.) of the fine and coarse aggregate was 2.82 and 6.73, respectively. Meanwhile, the steelmaking slag used in this study had a high value of blaine and strong water-absorbing properties [20]. These properties increase the viscosity of the concrete, thereby reducing the fluidity. Therefore, to solve this problem, a polycarboxylate-based superplasticizer produced in Republic of Korea which had the density of 1.07 g/cm^3^ with dark brown color was incorporated into all mixes to improve workability.

**Figure 2:**
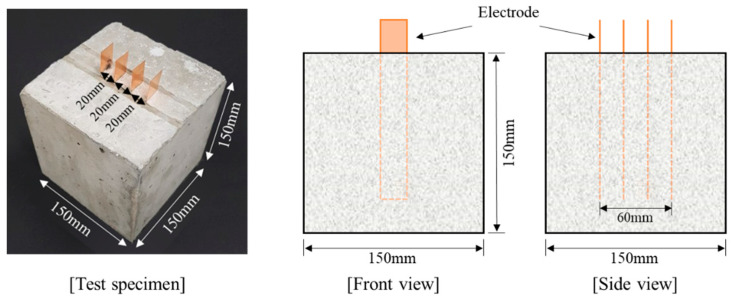
Dimensions of test specimen for evaluating electrical characteristics.

Setting, compressive strength, and splitting tensile strength tests were carried out to evaluate the strength characteristics of all the mixes. According to the method specified in ASTM International, setting test specimen was ∅150 mm × 150 mm in size, and the compressive and splitting tensile strength test specimens were fabricated into cylinders of ∅100 mm × 200 mm [21,22,23,24]. Concrete test specimens for assessing electrical characteristics were 150 mm cubes considering the maximum dimensions of the coarse aggregate, as shown in Figure 2 [4,12]. Copper plates which had dimension of 20 mm × 150 mm were used as electrodes, and four copper plates were embedded in the concrete at intervals of 20 mm. After casting the concrete into the mold, the test specimens were cured in a constant temperature and humidity room with a temperature of 20 ± 1 °C and a humidity of 60 ± 5%. After 14 days, half of test specimens were dried by oven at 60 °C for 72 h to determine the effect of the moisture present in the concrete on the electrical properties.

### 2.2. Measurement of Electrical Resistance and Self-Sensing Capacity

In general, electrical resistance can be measured with two- and four-probe methods. Although the two-probe method has a simpler circuit than the four-probe method, some studies have reported that the four-probe method is easier to control contact resistance than the two-probe method [2,25,26]. Therefore, the four-probe method was used in this study. In addition, when DC current is used in the current system, the resistance increases with time due to the polarization effect [27,28]. Therefore, AC current in the 100 kHz band was used to control the effects of the polarization [29,30].

**Figure 3:**
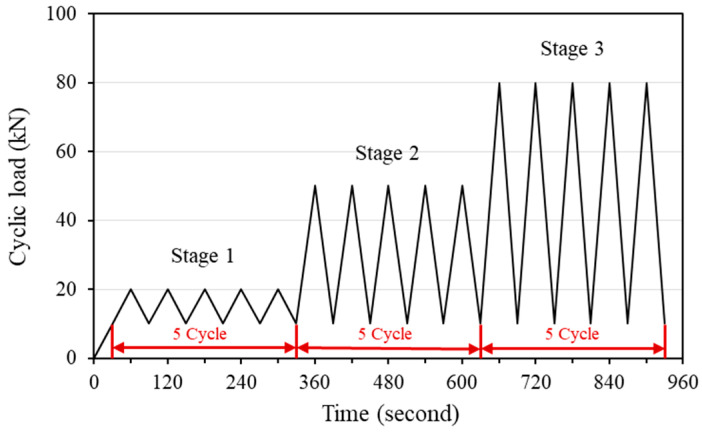
Cyclic loading protocol for the self-sensing test.

**Figure 4:**
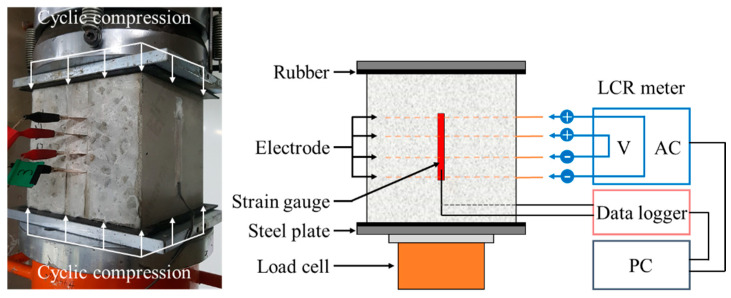
Test setup of self-sensing under cyclic compressions.

To evaluate the change of electrical resistance with age, the electrical resistance was measured at the designated age using an LCR meter (GWINSTEK, LCR-819, Taiwan, China). In addition, to assess the self-sensing capacity of concrete incorporated with steelmaking slag, a cyclic compressive load was applied to a 150 mm cubic test specimen using a universal testing machine (UTM/MTS, 815, Minneapolis, MN, USA) after 28 days from the casting. The steel plates were placed on both the upper and lower sides of the test specimen so that equal compressive forces were distributed to the test specimen under loading. In addition, to eliminate the effect of steel plates when measuring electrical resistance, an approximately 2-mm-thick piece of rubber that acts as an insulator was placed between the test specimen and the steel plates [4,31]. Concrete surface strain gauges were attached to both sides of the test specimen and self-sensing tests were performed to measure the strain change and the electrical resistance of concrete with repeated compressive loads. The cyclic compressive load was applied in the same way as the previous study [4] on concrete mixed with conductive materials. Three stages of load range were set and five cyclic loads were repeated for each load stage. The repeated loading protocol and test setup of self-sensing under cyclic compressive loads are shown in Figure 3 and Figure 4.

## 3. Experimental Results and Discussion

### 3.1. Strength Properties of Concrete Containing Steelmaking Slag

#### 3.1.1. Setting Times

To determine the setting times of all mixes, the penetration resistance stress was measured over time. Afterwards, the initial and final setting time were derived through the regression analysis using Equation (1), and the time points at which the penetration resistance stress satisfied 3.5 and 28.0 MPa were defined as initial and final setting, respectively.

*Log*(*PR*) = *a* + *bLog* (*t*) (1)

where *PR* is the penetration resistance stress in MPa, *t* is the elapsed time after mixing, and *a* and *b* are the regression constants.

**Figure 5:**
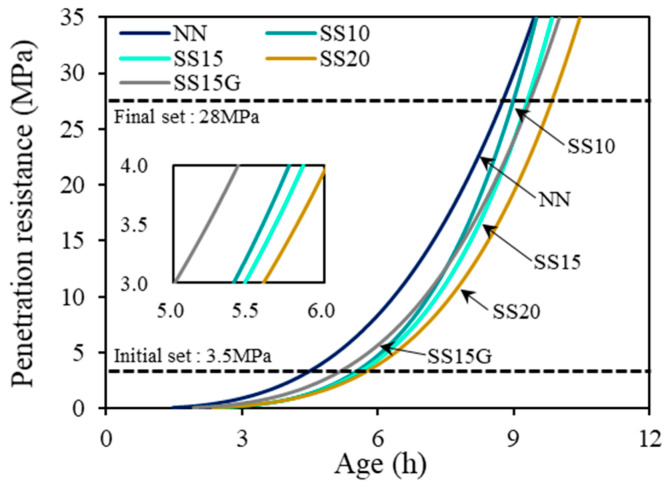
Initial and final setting curve.

The NN took 4.54 h (272 min) to reach the initial setting and 8.79 h (528 min) to reach the final setting. However, for mixes that contained any amount of steelmaking slag, initial and final setting of all the mixes occurred after 5 and 9 h respectively. As the replacement ratio of steelmaking slag increased, the hydration process tended to be more delayed, which was attributed to the decrease in cement usage due to replacement of steelmaking slag. In addition, steelmaking slag has a lower hydraulicity compared to cement, which is another reason for delay of setting times [16]. For these reasons, initial setting of SS10, SS15, and SS20 occurred 1.05 (63 min), 1.13 (68 min), and 1.28 h (77 min) later than NN, respectively. However, SS15G delayed 0.68 h (41 min) compared to NN, and its initial setting occurred faster than SS10. This is believed to be because the incorporation of gypsum influenced the hydration velocity. The calcium sulfate hemihydrate gypsum used in this study is a soluble gypsum, which easily absorbs the surrounding moisture and is thought to accelerate the hardening speed [32]. It is anticipated that the incorporation of gypsum may partially develop the reduction in the early strength of concrete caused by the low hydraulicity of steelmaking slag. Table 3 shows the test results of initial and final setting by regression analysis and Figure 5 is a representation of the setting behavior of the mixes.

#### 3.1.2. Compressive Strength

**Figure 6:**
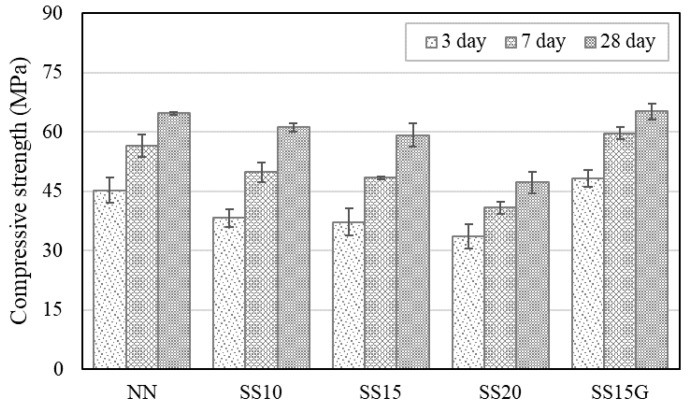
Test results of compressive strength at three different time points.

Figure 6 shows the results of compressive strength evaluation according to the replacement ratio of steelmaking slag. At the early age, when steelmaking slag was incorporated, the compressive strength was lowered, and as the replacement ratio increased, the strength difference from NN became larger. It is judged that steelmaking slag has lower hydraulicity than cement, and as the replacement ratio of steelmaking slag increased, setting time was delayed, resulting in a decrease in compressive strength [12,16]. The delay of hydration process due to the incorporation of steelmaking slag was demonstrated through the evaluation of setting time in this study. SS10 and SS15 were measured approximately 60 MPa at 28 days and showed compressive strength similar to NN, while SS20 reduced compressive strength by about 27% compared to NN. On the other hand, SS15G incorporating gypsum showed a different behavior from other mixes with any amount of steelmaking slag, and SS15G showed similar or higher strength compared to NN at all ages. This is because gypsum accelerated hydration and improved strength [33]. As a result, the incorporation of gypsum improved the drop in early compressive strength that occurred when steelmaking slag was incorporated, and the tendency shown in this study is consistent with the results of a study that confirmed that the strength of the concrete containing electric arc furnace oxidizing slag improved through the incorporation of gypsum [16].

#### 3.1.3. Splitting Tensile Strength

**Figure 7:**
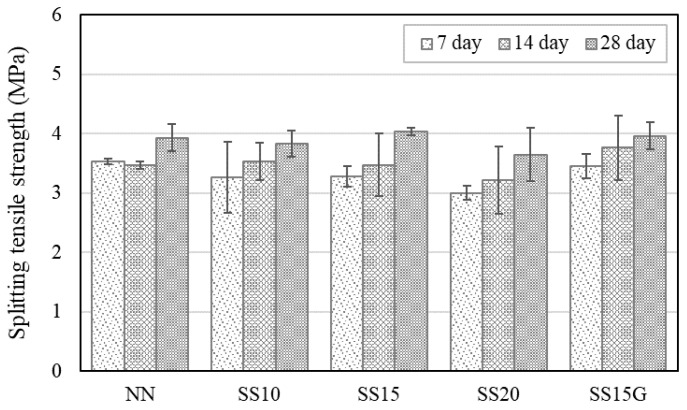
Test results of splitting tensile strength at three different time points.

The splitting tensile strength of concrete containing steelmaking slag also followed similar trends as those of compressive strength, which is shown in Figure 7. Incorporation of steelmaking slag lowered the splitting tensile strength, and, at seven days, SS10, SS15, and SS20 decreased in strength by 7.71%, 7.31%, and 15.12%, respectively, compared to NN. However, the decrease in strength by steelmaking slag was improved through the incorporation of gypsum, and, at 14 and 28 days, SS15G increased in strength by 8.30% and 14.06%, respectively, compared to NN.

### 3.2. Electrical Properties of Concrete Containing Steelmaking Slag

#### 3.2.1. Electrical Resistivity

Electrical resistivity is an indicator that can express the electrical properties of concrete. In this study, electrical resistance was measured at the designated age, and the electrical resistivity was derived by substituting it into Equation (2).

*ρ* = *RA*/*l* (2)

where *ρ* is the electrical resistivity, *R* is the electrical resistance measured by LCR meter, *A* is the contact area of the concrete with the electrode, and *l* is the distance between the electrodes.

**Figure 8:**
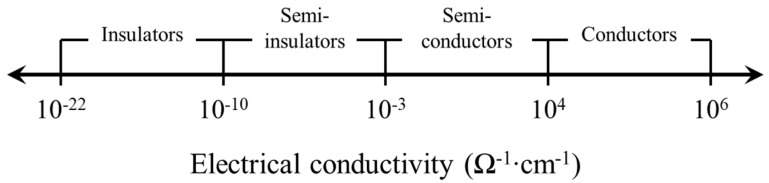
Scale of electrical conductivity [34].

Steelmaking slag is a well-known conductive material that has a large amount of iron oxide, which is the main ingredient of magnetite (Fe_3_O_4_), with the electrical conductivity of 100–1000 Ω^−1^ cm^−1^, and this is within the range of semi-conductors [34]. Figure 8 represents the range of electrical conductivity.

**Figure 9:**
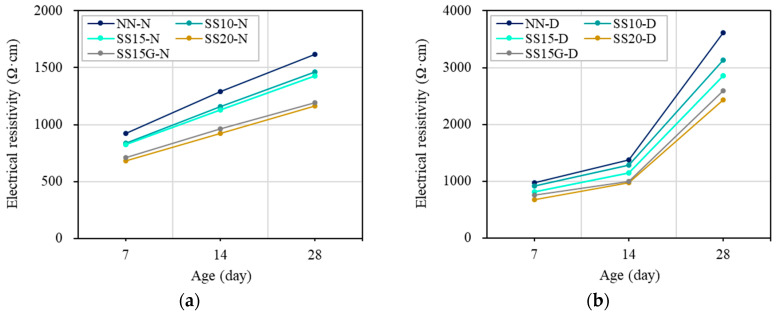
Electrical resistivity of concrete: (**a**) normal series; and (**b**) dry series.

**Figure 10:**
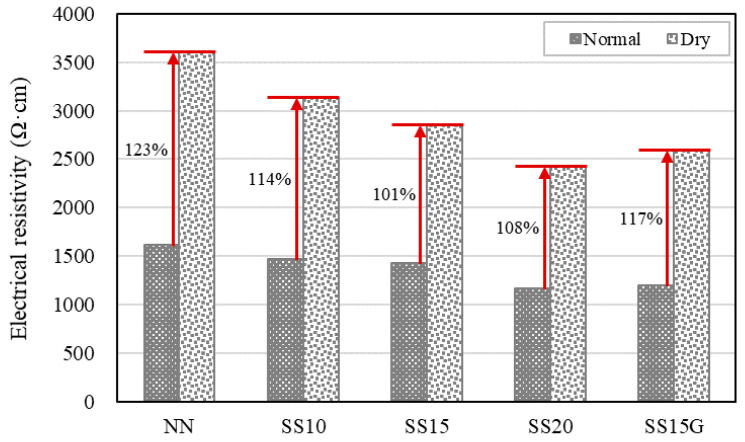
Increment of electrical resistivity at 28 days.

At 7 and 14 days, NN showed the highest electrical resistivity of all mixes. The incorporation of steelmaking slag decreased the electrical resistivity of concrete, and the conductivity of the concrete was shown to be better as the replacement ratio of steelmaking slag was increased. It is noteworthy that the incorporation of gypsum affects the electrical resistance of concrete. At seven days, SS15 had a difference in electrical resistivity of approximately 141.70 Ω·cm from SS20, but, when gypsum was incorporated, the electrical resistivity decreased by about 112.81 Ω·cm compared to SS15. This was because, as soon as the gypsum came into contact with the water in the concrete, the conductivity of the gypsum was dramatically improved and thereafter remained constant [35]. Due to these characteristics of gypsum, SS15G showed similar electrical resistivity as SS20. In addition, the electrical resistivity at 14 days for all mixes tended to be higher than the electrical resistivity of seven days. This is due to the evaporation of the pore water that assists the flow of current within the concrete as the age passes [9,29]. On the other hand, to determine the effect of the moisture present in the concrete on the electrical resistance, the concrete specimens were dried for a certain period after 14 days. Thereafter, at 28 days, the electrical resistances of the normal and dry specimens were measured. In the normal series, the electrical resistivity of all mixes at 28 days was distributed in the 1100–1700 Ω·cm, while the electrical resistivity in the dry series increased to 2400–3700 Ω·cm. The test specimen of all mixes was reduced by approximately 0.12–0.14 kg through drying, and NN, SS10, SS15, SS20, and SS15G of dry series increased electrical resistivity by 123%, 114%, 101%, 108%, and 117%, respectively, compared to normal series. The increase in electrical resistivity was greatest in NN, and it can be seen that the electrical resistance of NN depends on the moisture in concrete [4]. Figure 9 compares the electrical resistivity under replacement ratio of steelmaking slag between normal and dry series and Figure 10 shows the increment of electrical resistivity by drying.

#### 3.2.2. Electrical Piezoresistivity

Concrete subjected to external force changes electrical characteristics depending on the conductive material incorporated, and the electrical piezoresistivity of concrete can be evaluated by fractional change in resistivity (FCR). Equation (3) is an expression of FCR.

*FCR* = *ΔR*/*R_0_* = (*R_x_* − *R_0_*)/*R_0_* (3)

where *R_0_* is the electrical resistance at the beginning of the experiment and *R_x_* is the electrical resistance at the time to load is applied.

**Figure 11:**
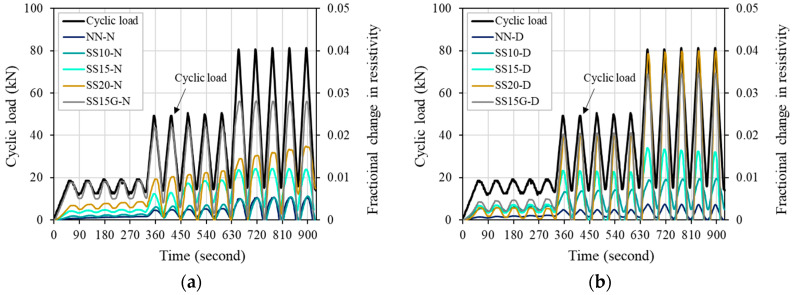
FCR response of concrete: (**a**) normal series; and (**b**) dry series.

In general, when a compressive load is applied to a test specimen, the electrical resistance in the test specimen decreases, resulting in a negative FCR. However, in this study, to smoothly compare the behavior of compressive load and FCR, the FCR was multiplied by a negative number (−1) so that a positive value appeared. Figure 11 shows the relationship between cyclic compressive load and FCR over time, and incorporating of steelmaking slag in both normal and dry series increased FCR. Through this, it can be seen that steelmaking slag improves electrical properties as a conductive material, as reported by Baeza et al. [13] and Lee, Le, and Kim [14].

In the normal series, it can be seen that NN increases the FCR in a straight line according to the change in load in Stage 1 (load range: 10–20 kN). In contrast, for SS10, SS15, SS20, and SS15G, the FCR fluctuated flexibly with changes in load. This result reflects that the steelmaking slag used in this study contains a large amount (approximately 40%) of iron oxide that was distributed evenly within the concrete matrix to form conductive paths. It is noteworthy that gypsum had a positive effect on FCR as well as electrical resistivity when repeated loads were applied, so that the best FCR behavior and FCR values were shown even at low load ranges. In Stage 2 (load range: 10–50 kN), the FCR fluctuated similarly with the change of the load for all mixes. This is because the distance between the steelmaking slag that was distributed inside the concrete matrix decreased as the maximum load increased compared to Stage 1, thereby increasing the formation of conductive paths [12,36]. The increase in compressive load decreased the potential barrier, and, as a result, the movement of electrons may have become smooth, and the variation in electrical resistance increased [30,36]. As the replacement ratio of the steelmaking slag increased, the FCR also tended to increase. As a result, the conductive path in the concrete was improved with the increase in the proportion of steelmaking slag. In Stage 3 (load range: 10–80 kN), similar behavior to Stage 2 was observed, and the FCR fluctuated similarly with the change in load. As the replacement ratio of the steelmaking slag increased, a larger difference and higher FCR were observed, and the maximum FCR for SS15 in Stage 3 was approximately 0.012. The FCR increased by 43.45% and 131.24% for SS20 and SS15G compared to SS15, respectively, and SS15G had the best self-sensing characteristics in all stages. In the case of SS20, the FCR value gradually increased as the loads were repeated in Stages 2 and 3. This is because the distribution location of the steelmaking slag that previously formed the conductive path was affected by the increase of the maximum load [4,30].

**Figure 12:**
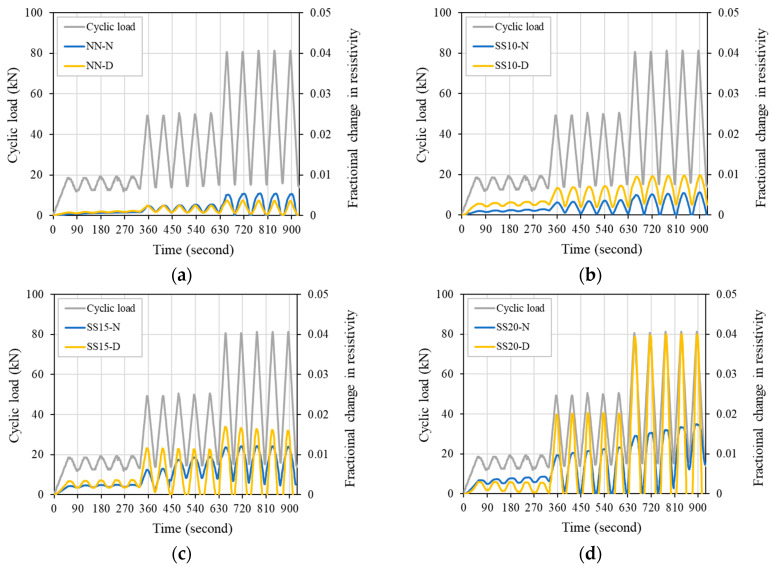

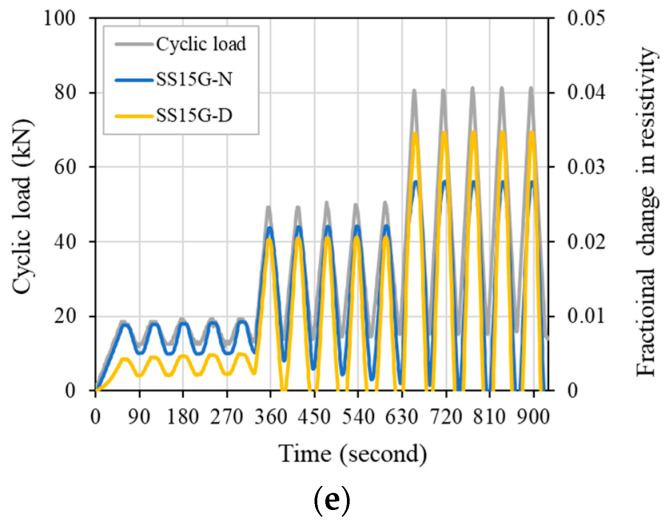
Moisture effect on FCR response of concrete: (**a**) NN; (**b**) SS10; (**c**) SS15; (**d**) SS20; and (**e**) SS15G.

After casting concrete, the internal moisture decreases with time, and finally it becomes dry condition. Decreasing moisture inside concrete increases electrical resistivity and degrades electrical properties. Therefore, in this study, the FCR of concrete was evaluated by drying the test specimens at a certain temperature for a specific period of time to exclude the effect of moisture in the concrete on the electrical properties [37]. In the dry series, unlike the normal series, all mixes that contained steelmaking slag fluctuated flexibly according to the change in load. In Stage 2, as the replacement ratio of steelmaking slag increased, a larger FCR value appeared, and SS20 and SS15G showed similar FCR values of about 0.02. In Stage 3, the FCR difference of the mixes was clearly revealed as the maximum load increased, and the maximum FCR for SS10, SS15, SS20, and SS15G compared to NN increased by 163.55%, 356.11%, 972.64%, and 831.26%, respectively. Figure 12 shows the effect of evaporation of moisture from drying specimen on concrete with steelmaking slag. Except for NN, in Stages 2 and 3, the dry series showed a relatively higher FCR value compared to the normal series. The maximum FCR of SS15, SS20, and SS15G increased by 40.10%, 129.68%, and 23.70%, respectively. Through comparison of normal series and dry series, it can be seen that moisture in concrete dominates the electrical piezoresistive characteristics of concrete.

#### 3.2.3. Electrical Sensitivity

The electrical sensitivity of concrete can be evaluated with a gauge factor (GF). In this study, the concrete surface strain and electrical resistance changes of the concrete were measured by applying the repeated compressive loads, and the GF was calculated by substituting them into Equation (4).

*GF* = *FCR*/*ε* (4)

where *ε* is the strain of the concrete surface.

**Figure 13:**
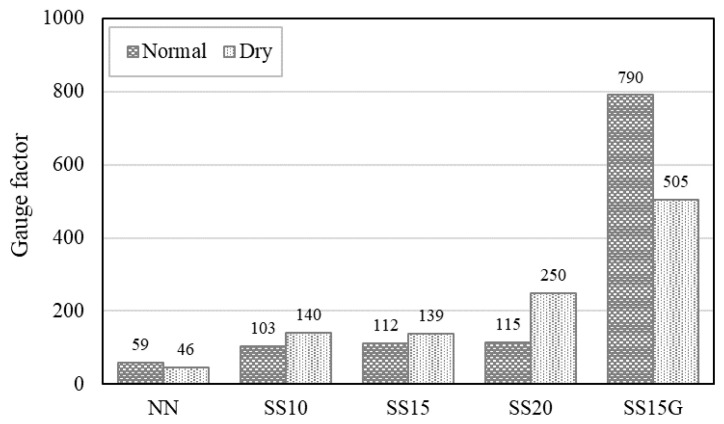
Comparison of gauge factor of concrete with steelmaking slag.

Figure 13 represents the GF of the all mixes. Both the normal and dry series showed a trend that the GF increased as the replacement ratio of the steelmaking slag increased. Similar to the results for electrical resistivity and electrical piezoresistivity, this result also indicates that the conductive path in the concrete matrix improved with an increase in the ratio of the steelmaking slag. In the normal series, the GF for SS10, SS15, SS20, and SS15G increased by 74.75%, 90.61%, 95.79%, and 1241.82% compared to NN. For dry series, SS10, SS15, SS20, and SS15G increased the GF by 206.18%, 203.93%, 446.62%, and 1005.60% compared to NN, respectively. Here, SS15G has 585.35% and 102.26% larger GF values in the normal and dry series, respectively, compared to SS20. SS15G showed similar electrical characteristics compared to SS20, but SS15G had superior resistance to external force compared to SS20. Due to these characteristics, SS15G showed the largest gauge factor value among all mixes.

**Figure 14:**
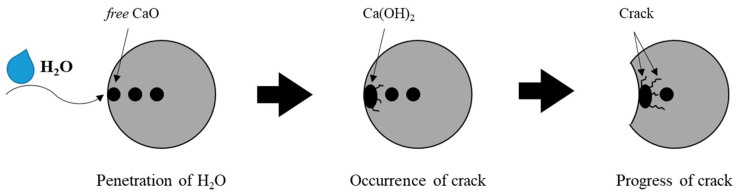
Cracking process of steelmaking slag by free CaO.

In general, the conductivity of concrete is affected by the moisture in the concrete. Demircilioglu et al. [38] and Song and Choi [39] showed that, when the moisture in the concrete decreases, it affects the piezoresistivity of the concrete, reducing the GF of the concrete. In this study, dried specimens of NN and SS15G, as previously reported, showed lower GF, whereas SS10, SS15, and SS20 increased GF when specimen was dried. This is considered to be the effect of free CaO, an expansive compound in steelmaking slag. As the compressive load is repeated, the moisture in the concrete comes into contact with free CaO, and continuous volume expansion results in the occurrence of micro cracks. These cracks in the matrix gradually grow under repeated loads, and this phenomenon ultimately affects the electrical properties of concrete, breaking or rebuilding the conductive path in the matrix [3,22]. Normal series specimens contained more moisture in concrete than dry series, so that SS10, SS15, and SS20 of normal series were affected by FCR, and the GF was finally derived to be low. When the specimens were dried, the GF of SS10, SS15, and SS20 increased by 37, 27, and 135, respectively. Figure 14 depicts the progress of crack due to expansion of free CaO.

## 4. Conclusions

In this study, the effect of the replacement ratio of steelmaking slag on the electrical properties of concrete was evaluated and the moisture in concrete was also considered. The conclusions drawn from this study are as follows:

- Steelmaking slag delayed hydration process, and, when steelmaking slag was incorporated, the strength decreased. However, it was confirmed that the reduction of the strength of the concrete containing steelmaking slag could be improved through the incorporation of gypsum.
- The electrical conductivity of steelmaking slag is included in the range of semi-conductors, and the electrical resistivity decreased as the replacement ratio of steelmaking slag increased. In addition, the incorporation of gypsum improved the conductivity of the concrete. On the other hand, the electrical resistivity is dependent on the moisture in the concrete, and, when the concrete specimen was dried, the electrical resistivity increased about two times or more.
- The iron oxide in the steelmaking slag formed a conductive path in the concrete, and the reduction of the potential barrier due to the increase in the cyclic compressive load increased the FCR. The dry series showed higher FCR values than the normal series, and, when the specimen was dried, the maximum FCRs of SS15, SS20, and SS15G increased by 40.10%, 129.68%, and 23.70%, respectively. Through this, it could be seen that the electrical piezoresistivity of concrete was affected by moisture.
- The GF improved as the replacement ratio of the steelmaking slag increased. In general, when the moisture in concrete decreases, the GF decreases; however, the mixes incorporating only steelmaking slag showed the opposite trend. This was because moisture in concrete came into contact with free CaO and caused cracks, which affected the conductive path in concrete. When the specimens were dried, the GF of SS10, SS15, and SS20 increased by 37, 27, and 135, respectively.

## Figures and Tables

**Table 1:** Mix design of concrete specimens.

Mix	W/B *	By Cement Weight Ratio				SS ** (%)	G *** (%)	SP **** (%)
		Water	Cement	Fine Aggregate	Coarse Aggregate			
NN	0.325	0.32	1.00	1.25	1.86	-	-	0.19
SS10	0.325	0.36	1.00	1.39	2.06	10	-	0.19
SS15	0.325	0.38	1.00	1.48	2.18	15	-	0.19
SS20	0.325	0.41	1.00	1.57	2.32	20	-	0.19
SS15G	0.325	0.38	1.00	1.48	2.18	15	2	0.19

W/B, water to binder (cement + ss) ratio; SS, steelmaking slag; G, gypsum. * W/B is calculated by dividing water from binder. ** Replacement ratio to cement weight of NN. *** Additive ratio to binder weight. **** Incorporation ratio to binder weight.

**Table 2:** Chemical compositions and physical properties of binder.

Composition % (Mass)	Cement	SS
SiO_2_	23.0	14.2
CaO	63.0	22.1
Al_2_O_3_	6.5	11.1
T-Fe *	3.0	39.9
MgO	2.0	3.33
SO_3_	1.9	0.02
MnO	-	5.59
TiO_2_	-	0.69
Density (g/cm^3^)	3.15	3.96
Blaine (cm^2^/g)	3413	4893

SS, steelmaking slag. * T-Fe, FeO, Fe_2_O_3_.

**Table 3:** Regression results of initial and final setting.

Mix	Initial Set (h)	Final Set (h)	a	b	R^2^
NN	4.54	8.79	−1.524	3.147	0.956
SS10	5.59	9.03	−2.697	4.336	0.989
SS15	5.67	9.33	−2.609	4.182	0.988
SS20	5.82	9.88	−2.464	3.932	0.993
SS15G	5.23	9.40	−2.001	3.544	0.911

a and b, regression coefficients; R^2^, coefficient of determination.
